# Draft Genome Sequence of *Streptomyces viridochromogenes* Strain Tü57, Producer of Avilamycin

**DOI:** 10.1128/genomeA.00384-13

**Published:** 2013-06-20

**Authors:** Björn A. Grüning, Anika Erxleben, Anna Hähnlein, Stefan Günther

**Affiliations:** Institute of Pharmaceutical Sciences, Pharmaceutical Bioinformatics, University of Freiburg, Freiburg, Germanya; Center for Biological Systems Analysis (ZBSA), University of Freiburg, Freiburg, Germanyb

## Abstract

Here we present the draft genome sequence of *Streptomyces viridochromogenes* Tü57. This strain is a producer of avilamycin A, an oligosaccharide antibiotic from the orthosomycin group, which is active against Gram-positive bacteria.

## GENOME ANNOUNCEMENT

*Streptomyces viridochromogenes* strain Tü57 is a producer of avilamycin A, a potent antibiotic against Gram-positive pathogenic bacteria. Avilamycin is an oligosaccharide antibiotic which belongs to the orthosomycin group (1). Members of this group show high antibacterial activity against glycopeptide-resistant enterococci, penicillin-resistant streptococci, and methicillin-resistant staphylococci (2–4). Avilamycin A consists of a hepta-saccharide side chain and a polyketide-derived dichloroisoeverninic acid as aglycone. Several genes from the secondary metabolite gene cluster which encodes the biosynthesis of avilamycin A have been characterized previously (5–7).

The whole-genome shotgun sequence was determined by Roche/454 GS FLX titanium pyrosequencing technology (8). Generation of a paired-end library consisting of 855,621 reads was performed, which corresponds to 17-fold coverage. All reads were assembled using Newbler 2.5.3, and 379 contigs were obtained. The genome of *Streptomyces viridochromogenes* Tü57 is composed of 9.7 Mbp, with an average G+C content of 71%.

For the analysis of the *S. viridochromogenes* Tü57 genome, open reading frames were predicted by Glimmer3 using a specific *Streptomyces* training set (9). Genome annotation was performed by applying an in-house-developed Galaxy-based automatic genome annotation pipeline for *Streptomycetes*. In order to determine putative secondary metabolites, prediction of gene clusters was performed using antiSMASH (10). The analysis revealed that 846 genes belong to 33 secondary metabolite gene clusters. From those, we identified two polyketide synthase type I (PKSI) gene clusters, one PKSII, two PKSIII, 9 nonribosomal peptide synthetase (NRPS), two hybrid NRPS-PKSI, three terpene, and 14 other gene clusters. Furthermore, the analysis of nontranslating genes predicted at least 68 tRNAs and 1 tmRNA on the genome (11, 12).

### Nucleotide sequence accession number.

This whole-genome shotgun project has been deposited at DDBJ/EMBL/GenBank under the accession number AMLP00000000. The version described in this paper is the first version.
